# Red Cell Distribution Width Has a Negative Prognostic Role in Dogs with Myxomatous Mitral Valve Disease

**DOI:** 10.3390/ani11030778

**Published:** 2021-03-11

**Authors:** Carlo Guglielmini, Chiara Martina Valentini, Barbara Contiero, Carlotta Valente, Helen Poser

**Affiliations:** Department of Animal Medicine, Production and Health, University of Padova, Viale dell’Università 16, 35020 Legnaro, Italy; chiaramartina.valentini@studenti.unipd.it (C.M.V.); barbara.contiero@unipd.it (B.C.); carlotta.valente@phd.unipd.it (C.V.); helen.poser@unipd.it (H.P.)

**Keywords:** canine, cardiac biomarker, cardiovascular disease, echocardiography, hematology, prognosis

## Abstract

**Simple Summary:**

The red cell distribution width (RDW) is a simple and inexpensive laboratory parameter that reflects the difference in size of the red blood cells (also known as anisocytosis) and is conventionally used in a clinical setting for the differential diagnosis of anemias. Nonetheless, recent studies have demonstrated that anisocytosis is commonplace in many non-hematological human disorders and an increased RDW has been associated with a negative prognosis in patients with different cardiovascular diseases. In dogs, no studies have evaluated the prognostic role of RDW with myxomatous mitral valve disease (MMVD). The present study evaluates clinical, echocardiographic, and laboratory parameters, including RDW, with a cohort of dogs with MMVD and followed up on for more than one year. We sought to evaluate if RDW acts as an independent prognostic marker for negative outcomes in dogs with MMVD with or without concurrent non-cardiac diseases.

**Abstract:**

Red cell distribution width (RDW) is a quantitative measurement of anisocytosis. This hematological parameter is an important prognostic biomarker for different cardiovascular disorders in humans but its influence on survival has been poorly investigated in dogs with cardiovascular disease. The RDW and various clinical, complete blood count, serum biochemical and echocardiographic variables were retrospectively investigated in 146 client-owned dogs with myxomatous mitral valve disease (MMVD) at various disease stages, with or without concurrent diseases and treatment. Laboratory variables, including RDW, urea, and white blood cell (WBC), in addition to the echocardiographic variable left atrium to aorta ratio were found to be independent predictors of all-cause mortality at six months in a multivariable Cox proportional hazards regression model. In particular, the hazard ratio of RDW was 1.203 (95% confidence interval = 1.045–1.384; *p* = 0.010). The negative effect of increased RDW on outcome was confirmed using Kaplan–Meier curve analysis. The results of this study indicate that RDW acted as an independent predictor of negative outcome in dogs with MMVD.

## 1. Introduction

The red cell distribution width (RDW) is an index of the heterogeneity of circulating red blood cell size, conventionally known as anisocytosis, and is a component of standard complete blood count (CBC) that is commonly calculated by modern hematology analyzers [1,2]. RDW, in addition to mean corpuscular volume (MCV), has been historically used for the evaluation of hematological disorders in humans and dogs, in particular for the differential diagnosis of anemias [1,2,3,4]. However, several clinical studies have shown that anisocytosis may be associated with other systemic diseases including gastrointestinal disorders, acute pancreatitis, chronic kidney disease, cancer, and cardiovascular disease in humans [2]. Hence, an increased RDW is commonplace in the above non-hematological disorders and has demonstrated a negative prognostic significance in patients with coronary artery disease, cerebrovascular and other peripheral artery disease, atrial fibrillation (AF), pulmonary hypertension (PH), and heart failure (HF) [2,5,6,7,8,9,10]. For these reasons, RDW appears to be an inexpensive, independent, and powerful prognostic marker in a broad population of cardiovascular patients [2,10].

In dogs, some retrospective studies evaluated RDW in animals with several non-hematological disorders including myxomatous mitral valve disease (MMVD), PH, heartworm disease, acute trauma, and different endocrine, neurological, gastrointestinal, respiratory, and renal diseases [11,12,13,14,15,16]. MMVD is the most common canine cardiac disease and conflicting results have been reported in two successive studies that evaluated RDW in affected dogs compared with clinically healthy dogs [11,15]. Furthermore, one study evaluated the prognostic value of different hematologic variables in dogs with acute trauma, but failed to demonstrate an association between RDW and survival in these animals [14]. To the author’s knowledge, no study has specifically evaluated the prognostic value of RDW in dogs with cardiac disease. Therefore, this study investigates the prognostic role of RDW in dogs with MMVD. We seek to determine if RDW is an independent prognostic marker of negative outcomes in dogs with MMVD with or without concurrent noncardiac diseases.

## 2. Materials and Methods

This retrospective cohort study was based on the retrieval of clinical data available in the database of the Veterinary Teaching Hospital of the University of Padua (VTH-UP). All the owners of dogs included in the study signed a written consent, which stated the diagnostic procedures and the potential use of the clinical data for research purposes.

### 2.1. Animals

Medical records of dogs diagnosed with MMVD at the VTH-UP from January 2014 to July 2019 and with an available CBC and serum biochemical profile were retrospectively reviewed. Laboratory exams were carried out within a maximum of 72 h from diagnosis, which was based on combined clinical (that is, left apical systolic heart murmur), electrocardiographic, radiographic, and echocardiographic findings. Transthoracic echocardiography, two-dimensional (2D) real time, M-mode, and echo-Doppler, was performed according to established guidelines [17] by two experienced operators (CG and HP) using an ultrasound unit (CX50, Philips, Eindhoven, Netherlands) and different phased array transducers. Continuous ECG monitoring was also employed. Dogs with echocardiographic features characteristic of MMVD, namely thickened, knobby or prolapsing mitral valve leaflets on 2D echo, mitral regurgitant blood flow on color mapping, and without other concurrent congenital or acquired cardiac diseases were included. Dogs with other concomitant noncardiac diseases were also included. Additional clinical information included the presence of AF, PH, concurrent diseases, and previous use of any drug for the treatment of cardiac or noncardiac disease. Dogs were also classified according to the more recent guidelines of the American College of Veterinary Internal Medicine (ACVIM) in four disease stages of MMVD. These included: stage B1 (that is, asymptomatic dogs with valvular disease but without cardiac remodeling); stage B2 (that is, asymptomatic dogs with cardiac remodeling involving both the left atrium (LA) and left ventricle (LV) evidenced by cardiac imaging); stage C (that is, symptomatic dogs with at least one present or past episode of HF); and stage D (that is, symptomatic dogs with HF refractory to standard therapy) [18].

### 2.2. Echocardiographic Examination

The measurement of LV internal diameters was carried out from M-mode images obtained from the right parasternal short axis view at chordal level. Normalization for the effect of the BW of the measured LV diameters was then obtained by applying previously published equations [19]: normalized LV diastolic diameter (LVDDn) = LV diastolic diameter/[BW^0.294^] and normalized LV systolic diameter (LVSDn) = LV systolic diameter/[BW^0.315^]. The diameters of the LA and aortic root (Ao) were measured at the first frame after aortic valve closure (that is, early diastole) from 2D images derived from the right parasternal short axis view at the level of the heart base, as previously described [20,21] and the LA-to-Ao ratio (LA/Ao) was then calculated. Trans-mitral blood flow was recorded from the left parasternal apical four-chamber view by positioning the sample volume of pulsed-wave Doppler interrogation at the tips of the mitral valve leaflets and measurement of the peak velocity of early diastolic blood flow (E-max) was then obtained. The mean value of three consecutive measurements of each echocardiographic and echo-Doppler variable was used for successive analysis.

The presence of PH was considered for all dogs without echocardiographic and Doppler evidence of any obstruction of the right ventricular outflow but with measurable tricuspid regurgitation (TR) with a maximum velocity ≥ 3.4 m/s on continuous-wave Doppler interrogation, and corresponding to a peak systolic pressure gradient ≥ 46 mmHg [22].

### 2.3. Blood Analysis

Complete blood count and serum biochemical analyses were carried out on blood samples obtained from dogs that had fasted for at least 12 h. The hematologic variables, including RDW, were measured using a commercial, automated CBC analyzer (Advia 120, Hematology system, Siemens, Munich, Germany) that was used to previously validate canine hematology [23]. Serum biochemical variables were measured using a commercial analyzer (AU 400, Mishima Olympus, Shizuoka, Japan). The internal quality controls provided by the manufacturers “Test Point Normal Control” and “Normal and Pathologic” were daily performed for hematology and clinical biochemistry analysis, respectively. The external quality control was carried out weekly for both analyzers using human control material (Bio-Rad Laboratories, Segrate, Italy) and according to EQA-RQAS (External Quality Assessment—Randox International Quality Assessment Scheme) monthly. The reference interval for RDW was 13.3–14.9%.

### 2.4. Statistical and Survival Analysis

Data analysis was carried out using different commercially available statistical software programs (SAS version 9.3, SAS Institute Inc., Cary, NC, USA; MedCalc version 12.6.1.0, MedCalc Software, Ostend, Belgium). Normal distribution of clinical, echocardiographic, and laboratory variables was assessed using the Shapiro–Wilk’s test. Normally and non-normally distributed data were reported as mean ± standard deviation and median and range, respectively. The categorical variables were presented as frequency counts and percentages. The Student’s t-test and the Mann–Whitney test were used to compare data of normally and non-normally distributed variables, respectively, with dogs divided in two groups: animals dead by six months and still alive after six months from the time of diagnosis. The chi-square test was applied to evaluate any difference between the categorical variables.

The association between all variables and RDW or survival after six months were investigated using the Spearman rank correlation analysis. Survival data were derived from the VTH-UP database or through telephone calls with the referring veterinarians or dogs’ owners. Dogs were classified as still alive, dead from all-cause mortality, or lost to follow up when no further data were available after presentation. Time in days from inclusion (i.e., diagnosis of MMVD) to death (survival time) or to the telephone interview for dogs still alive (follow-up time) was annotated. Right-censoring was applied for dogs still alive at the end of the observational period. Kaplan–Meier curves were constructed for each tercile of RDW on survival time.

Univariate Cox proportional hazards regression analysis was performed to determine whether a significant relationship existed between clinical, echocardiographic, and laboratory variables, including RDW, and survival. The endpoint was death within six months from diagnosis due to all-cause mortality. The hazard ratio (HR) and 95% confidence intervals (CI) were calculated considering one-unit change or the level of interest for the continuous or the categorical variables, respectively. Variables showing *p* < 0.1 in the univariate analysis were then tested in a multivariable Cox proportional hazards regression model using a manual forward selection method to determine the independent predictor’s effect on survival.

The level of significance was set at *p* values < 0.05 for all analyses.

## 3. Results

### 3.1. Animals

The inclusion criteria was met by 146 dogs (60.3% male and 39.7% female) of different breeds, including 57.5% mixed breed dogs. Their mean age was 143 ± 35 months and the median BW was 9 kg (2–38 kg). At inclusion, 65 dogs (44.5%) were receiving no treatment and 81 dogs (55.5%) one or more drugs for MMVD, concomitant noncardiac disease, or both. Table A1 shows an overview of the type of treatment and drugs administered. Thirty-one dogs (21.2%) had concomitant non-cardiac disease including neurological (10 cases), respiratory (9 cases), neoplastic (4 cases), genital (3 cases), endocrine and urinary (2 cases each), and traumatic disorders (1 case). Thus, 56 dogs (38.4%) had no other concomitant disease and were receiving no treatment; 44 dogs (30.1%) were taking one or more drugs for MMVD; 16 dogs (11%) were taking one or more drugs for MMVD and a concomitant disease or had a concomitant not-treated disease; and 30 dogs (20.5%) were taking one or more drugs only for concomitant disease or had a concomitant not-treated disease. Ninety-five dogs (65.1%) were diagnosed with compensated MMVD (stage B1 and B2) and 51 dogs (34.9%) had decompensated MMVD (stage C and D). Atrial fibrillation was diagnosed in five dogs (3.4%). One hundred and twenty dogs had recordable TR (82.8%) and PH was diagnosed in 18 of these (12.3%). In 26 dogs (17.8%) neither TR nor pulmonic insufficiency was detected and, thus, the pulmonary pressure status could not be assessed. Among the 146 enrolled dogs, 34 (23.3%) had RDW greater than the upper reference limit of 14.9%.

At the end of the follow up time, 41 dogs (28.1%) were dead by six months from diagnosis (median survival time 70 days, range 1–176 days) and 105 dogs (71.9%) were still alive after six months from diagnosis (median follow-up time 575 days, range 190–2000 days).

### 3.2. Laboratory and Echocardiographic Variables

A summary of the characteristics of the 146 dogs in the study cohort, including their clinical, laboratory and echocardiographic data grouped according to death or survival at six months from diagnosis is shown in Table 1. In dogs dead by the six month median, serum urea and white blood cell (WBC) was significantly higher compared to that of dogs alive at 6 months (*p* = 0.001 and *p* < 0.001, respectively). Furthermore, dogs dead by 6 months had a significantly higher mean LA/Ao and E-max compared to that of dogs still alive (*p* = 0.004 and *p* = 0.027, respectively). Finally, a higher percentage of dogs without any treatment at the time of diagnosis was still still alive at six months from enrollment compared to dogs dead by six months (*p* = 0.002).

### 3.3. Correlation and Survival Analysis

Results of Spearman’s correlation to RDW and survival at six months for clinical, laboratory and echocardiographic variables are shown in Table 2. Age and serum total protein were significantly and positively, but weakly correlated with RDW. Furthermore, decompensated HF (that is, ACVIM stage C and D), red blood cell count, hemoglobin, hematocrit, MCV, and LVDDn were significantly and negatively, but weakly correlated with RDW. Moreover, age, decompensated HF, cardiac and mixed treatment, as well as urea, WBC, RDW, LA/Ao, LVDDn, E-max, and TR Vmax were significantly and negatively, but weakly correlated to survival at six months.

Results of Cox proportional hazard univariate analysis for predictors of all-cause mortality at six months are shown in Table 3. Twelve variables, including clinical (that is, age, previous cardiac treatment, decompensated HF, and presence of AF), laboratory (that is, urea, WBC, and RDW), and echocardiographic variables (that is, LA/Ao, LVDDn, LVSDn, E-max, and TR Vmax) were significant predictors of poor outcome in the univariate analysis.

Of the 12 variables that were significant in the univariate model, only four remained significant in the final multivariable model, namely urea, LA/Ao, RDW, and WBC (Table 4). Although urea was the strongest predictor of mortality, based on the chi-square statistic in the final model, LA/Ao had the highest HR of death (HR = 1.892, 95% CI = 1.232–2.905; *p* = 0.004) followed by RDW (HR = 1.203, 95% CI = 1.045–1.384; *p* = 0.010).

**Figure 1:**
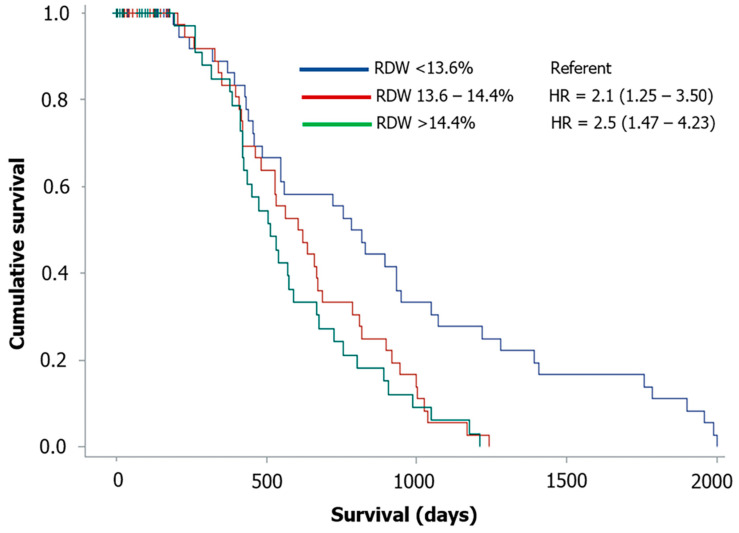
Kaplan–Meier survival curves comparing the survival time for all-cause mortality in 146 dogs with myxomatous mitral valve disease divided according to red cell distribution width (RDW) terciles. HR: hazard ratio.

Figure 1 shows the Kaplan–Meier plots for RDW divided by terciles. Dogs with RDW in the second (RDW = 13.6–14.4%) and third tercile (RDW > 14.4%) had significantly lower survival time and higher risk of death (HR = 2.09 and 2.5, 95% CI = 1.25–3.50 and 1.47–4.23; *p* = 0.005 and *p* < 0.001, respectively) when compared to dogs with RDW in the first tercile (RDW < 13.6%).

## 4. Discussion

The results of the present study showed that increased RDW is significantly associated with a negative outcome in dogs with MMVD, with or without concurrent diseases and treatment, independently from anemia or concomitant changes of MCV. Additional predictors of negative outcome were WBC and blood urea concentration and echocardiographic LA/Ao. Some observational studies have investigated the RDW in dogs with cardiovascular and other systemic diseases so far, but none of them has described an association between RDW and survival in dogs. Conversely, several studies showed that RDW is a strong and independent predictor of increased morbidity and mortality in human patients suffering from systemic diseases and cardiovascular disease [2,5,6,7,8,9,10]. Our findings highlight the importance of evaluating some simple laboratory parameters such as RDW in dogs with MMVD. Standardization of erythrocyte sizing can be an issue in both human and veterinary hematology [23,24]. In this study we used a bench-top hematology analyzer considered the “gold standard” for canine hematology [23]. However, the reference ranges of hematology variables, including RDW, can vary when using different laboratory instruments [23,24].

The preliminary analysis showed that some laboratory (that is, urea and WBC) and echocardiographic variables (that is, LA/Ao and E-max) were significantly higher in the group of dogs with MMVD that died within 6 months from diagnosis when compared to dogs with longer survival times. The same variables were also negatively correlated with survival after 6 months and other clinical (that is, age, advanced ACVIM stage, cardiac and mixed treatment), laboratory (that is, RDW) and echocardiographic variables (that is, LVDDn and TR Vmax). Furthermore, the results of the Spearman correlation analysis showed that RDW was positively correlated with age, suggesting a slight increase in anisocytosis with aging. In humans, RDW tends to increase in parallel with age [25] and elderly subjects have higher anisocytosis values [26]. In dogs, conflicting results have been reported regarding this association. No specific increase of RDW was found in a study that described the “anemia of the elderly” in elderly dogs with reduced hematocrit, MCV, and serum iron [27]. Furthermore, other previous studies specifically focused on the RDW in dogs with different diseases but failed to show evidence for age-associated changes with RDW [11,15]. However, another study carried out on dogs with PH reported a positive but moderate correlation between RDW and age [13]. These conflicting findings can be due to differences in aims and animal selection among different studies. The effect of age on RDW deserves further prospective investigation, particularly when studying age-associated disease of dogs like MMVD. Conversely, other negative correlations of RDW observed in the present study, such as those with RBC, hematocrit, hemoglobin, and MCV were consistent with the results of several other investigations both in dogs [11,12,13,14,15] and humans [7,28,29], which further confirmed the association between RDW and regenerative anemia.

The univariate analysis identified different predictors of adverse outcome including clinical data, namely age, cardiac treatment, advanced ACVIM stage and presence of AF, and laboratory data, namely urea, WBC, and RDW, and echocardiographic variables, namely LA/Ao, LVDDn, LVSDn, E-max, and TR Vmax. Many of them, particularly the echocardiographic variables, have already been reported as predictors of poor prognosis in dogs with MMVD [30,31,32,33,34,35,36,37,38,39,40]. However, only urea, WBC, RDW and LA/Ao remained significant predictors of adverse outcome in the final multivariable model. Differences in the prognostic role of some variables in the present study compared to previous studies on dogs with MMVD may have several explanations. First, we used all-cause mortality as an end-point rather than the previously most commonly employed cardiac death. This choice was based on the advanced age of dogs with MMVD and the consequent frequent association with other comorbidities that have an influence on mortality. Second, we found many collinearities among different echocardiographic variables in our final multivariable model. Thus, only LA/Ao was retained in the model, similarly as done by other previous studies [30,31,36,39]. Third, we used only laboratory variables of CBC and serum biochemical profile, instead of specific cardiac biomarkers (for example, N-terminal pro-B-type natriuretic peptide and cardiac troponin I) that were evaluated in previous studies on dogs with MMVD [32,33]. Finally, AF, that had the highest HR in the univariate analysis, was not retained in the final multivariable model, likely because of both collinearity with some echocardiographic variables (for example, the LA/Ao) and the low prevalence of arrhythmia in dogs with MMVD [41], including those of the present study. Although urea was the strongest predictor of a negative outcome, the LA/Ao still remained the variable with the highest HR in our model, followed by RDW. In particular, per each 1% increase of RDW there was a 20% increase of death at six months in dogs used in this study. The negative role of increased RDW was further confirmed by Kaplan–Meier curve analysis showing that dogs with RDW in the second (that is, within our reference interval) and third terciles had more than twice the risk of death compared to those with RDW in the lowest tercile. Increased WBC and urea were also associated with an increased risk of death in dogs in this study. Increased WBC has been reported in dogs with cardiac disease and decompensated HF and likely reflects an inflammatory status associated with advanced cardiac disease [42,43,44]. Similarly, increased urea or creatinine concentration, or both has been described in dogs with MMVD with progressive increase of azotemia along with the severity of congestive HF functional classes [11,45,46], and a significant correlation between blood urea value and ACVIM stages was found in dogs with MMVD [47]. Furthermore, a recent study showed that azotemia, in addition to other clinical (for example, cardiac cachexia) and laboratory variables, was associated with shorter survival times in dogs with cardiac disease [48].

The negative effect of RDW on outcomes observed in the dogs of this study reflects similar results as reported in several studies conducted in human patients with cardiovascular disorders, metabolic disorders (for example, diabetes, kidney and liver disease), acute conditions (for example, acute poisoning, acute pancreatitis, and critically ill patients) and chronic conditions (for example, cancer, inflammatory bowel disease, and chronic obstructive pulmonary disease) [2,5,6,7,8,9,10]. Although convincing evidence has been proved about the clinical significance of RDW in different human disorders, the question if anisocytosis is a real risk factor or a simple epiphenomenon (e.g., a “marker”) of an underlying metabolic or biological imbalance remains open [2]. Even though a simple and unequivocal explanation of the effect of increased RDW on outcomes cannot be given so far, several biological and metabolic abnormalities associated with different human disorders can exert a considerable effect on erythropoiesis including oxidative stress, inflammation, poor nutritional status, dyslipidemia, erythrocyte fragmentation, decline of erythropoietin synthesis, and erythropoietin hyporesponsiveness [2]. Some of these abnormalities has been reported also in dogs with cardiac disease, including those affected by MMVD [42,48,49,50,51].

This study has several limitations because of the retrospective design. First, because only those dogs with MMVD and available CBC and biochemical profile were included, there was an inherent bias in case selection. Second, only a single evaluation of RDW was available for dogs included in this study and, therefore, the RDW could have changed over the time interval between measurement and death or follow up. However, we chose to consider a quite short time interval between laboratory analysis and the end-point for all-cause mortality to overcome this drawback. Third, only some laboratory variables of CBC and biochemical profile were considered in our analysis. The evaluation of other variables could have offered additional information regarding the pathophysiological role of different abnormalities potentially associated with anisocytosis. Furthermore, the presence of different concurrent diseases and their treatment was considered as a single variable instead of performing a single analysis for each of them. Finally, since the included dogs were either first visits of the VTH-UP or animals referred for diagnostic purposes that were then treated by the referring veterinarians, the effect of medical therapy during the follow-up could not be evaluated.

## 5. Conclusions

In conclusion, results of the present study showed that RDW, even within reference intervals, is an independent predictor of poor outcomes in dogs with MMVD. This information can be useful for veterinary cardiologists and offer new interesting prospective on the prognostic use of this inexpensive and readily available laboratory parameter that is also found in other canine disease conditions as previously demonstrated in humans. Further prospective longitudinal studies could confirm our preliminary results and offer additional explanation of the complex relationship between anisocytosis and adverse outcomes in dogs with cardiac and noncardiac diseases.

## Figures and Tables

**Table 1:** Clinical data for the total study cohort and comparisons between dogs that died by six months and those alive with more than six months of follow-up.

Variable	Total Cohort (*n* = 146)	Dead by 6 Months (*n* = 41)	Alive at 6 Months (*n* = 105)	*p*-Value
Age (month)	143 ± 35	152 ± 32	140 ± 36	0.057
Weight (kg)	9 (2–38)	8 (2–38)	9 (2–3)	0.979
Sex (F/M)	58/88	19/22	39/66	0.410
Pure breed (*n* [%])	62 (42.5)	22 (53.7)	40 (38.1)	0.128
No treatment (*n* [%])	56 (38.4)	8 (19.5)	48 (45.7)	0.002
Cardiac treatment (*n* [%])	44 (30.1)	16 (39.0)	28 (26.7)	0.223
Mixed treatment/disease (*n* [%])	16 (11.0)	7 (17.1)	9 (8.6)	0.294
Other treatment/disease (*n* [%])	30 (20.5)	10 (24.4)	20 (19.0)	0.637
ACVIM stage C + D (*n* [%])	51 (34.9)	20 (48.8)	31 (29.5)	0.051
AF (*n* [%])	5 (3.4)	2 (4.9)	3 (2.9)	0.458
PH (*n* [%])	18 (12.3)	6 (14.6)	12 (11.4)	0.812
Urea (mg/dL)	43 (16–202)	60 (21–202)	40 (16–138)	0.001
Total protein (g/L)	71.4 ± 7.8	70.3 ± 9.8	71.9 ± 6.9	0.266
Albumin (g/L)	29.7 ± 4.8	29.1 ± 5.8	30.3 ± 3.6	0.137
Creatinine (mg/dL)	1.05 (0.57–3.02)	1.09 (0.57–2.39)	1.05 (0.60–3.02)	0.318
WBC (10^3^/μL)	11.42 (4.85–48.54)	14.02 (6.05–48.54)	9.92 (4.85–39.31)	<0.001
RBC (10^6^/μL)	6.92 ± 1.01	6.96 ± 1.26	6.9 ± 0.9	0.737
Hemoglobin (g/dL)	16.2 ± 2.4	16.3 ± 2.9	16.1 ± 2.1	0.667
Hematocrit (%)	46.2 ± 6.8	46.7 ± 8.5	46.0 ± 6.1	0.606
RDW (%)	14.0 (12.1–21.8)	14.0 (12.2–18.9)	13.9 (12.1–21.8)	0.708
MCV (fL)	67.0 ± 4.3	67.4 ± 4.8	66.8 ± 4.2	0.499
Platelet (10^3^/μL)	361.2 ± 135.6	344.7 ± 149.2	367.6 ± 130.0	0.361
LA/Ao	2.07 ± 0.68	2.33 ± 0.67	1.97 ± 0.66	0.004
LVDDn	1.76 ± 0.37	1.83 ± 0.4	1.74 ± 0.36	0.219
LVSDn	0.98 ± 0.22	0.99 ± 0.24	0.97 ± 0.21	0.651
FS (%)	41.9 ± 9.6	42.1 ± 11.9	41.8 ± 8.6	0.875
E-max (cm/s)	104.0 ± 40.0	115.6 ± 37.9	99.3 ± 40.1	0.027
TR Vmax (m/s)	2.72 ± 0.73	2.89 ± 0.8	2.66 ± 0.69	0.115

*n*: number of dogs; ACVIM: American College of Veterinary Internal Medicine; AF: atrial fibrillation; PH: pulmonary hypertension; WBC: white blood cell; RBC: red blood cell; RDW: red cell distribution width; MCV: mean corpuscular volume; LA/Ao: left atrial to aortic root diameter ratio; LVDDn: left ventricular diastolic diameter normalized for body weight; LVSDn: left ventricular systolic diameter normalized for body weight; FS: fractional shortening; E-max: trans-mitral peak E-wave velocity; TR Vmax: peak velocity of tricuspid regurgitation.

**Table 2:** Spearman correlation coefficients between red cell distribution width (RDW) or survival at six months and clinical, laboratory and echocardiographic variables.

Variable	RDW	*p*-Value	Survival (d)	*p*-Value
Age (month)	0.206	0.013	−0.261	0.002
Weight (kg)	0.091	0.275	−0.092	0.268
Sex—Male	−0.047	0.575	0.039	0.638
Breed—Pure breed	0.059	0.480	−0.041	0.623
Cardiac treatment Vs. No Treatment	0.159	0.113	−0.359	<0.001
Mixed treatment/disease Vs. No Treatment	0.087	0.468	−0.301	0.010
Other treatment/disease Vs. No treatment	0.075	0.494	−0.151	0.164
ACVIM stage C + D	−0.211	0.011	−0.265	0.001
AF	0.031	0.707	−0.123	0.139
PH	0.101	0.226	−0.122	0.142
Urea (mg/dL)	0.109	0.191	−0.342	< 0.001
Total protein (g/L)	0.188	0.024	0.082	0.326
Albumin (g/L)	0.007	0.936	0.142	0.087
Creatinine (mg/dL)	0.031	0.712	−0.135	0.105
WBC (10^3^/μL)	−0.056	0.499	−0.388	<0.001
RBC (10^6^/μL)	−0.193	0.020	0.054	0.516
Hemoglobin (g/dL)	−0.329	< 0.001	0.051	0.540
Hematocrit (%)	−0.322	< 0.001	0.061	0.462
RDW (%)	NA	NA	−0.171	0.040
MCV (fL)	−0.277	0.001	0.030	0.718
Platelet (10^3^/μL)	0.119	0.152	−0.035	0.677
LA/Ao	−0.123	0.138	−0.352	< 0.001
LVDDn	−0.246	0.003	−0.175	0.034
LVSDn	−0.163	0.050	−0.144	0.082
FS (%)	−0.024	0.778	−0.015	0.861
E-max (cm/s)	−0.087	0.300	−0.282	0.001
TR Vmax (m/s)	0.094	0.306	−0.249	0.006
Survival (d)	−0.171	0.040	NA	NA

ACVIM: American College of Veterinary Internal Medicine; AF: atrial fibrillation; PH: pulmonary hypertension; WBC: white blood cell; RBC: red blood cell; RDW: red cell distribution width; MCV: mean corpuscular volume; LA/Ao: left atrial to aortic root diameter ratio; LVDDn: left ventricular diastolic diameter normalized for body weight; LVSDn: left ventricular systolic diameter normalized for body weight; FS: fractional shortening; E-max: trans-mitral peak E-wave velocity; TR Vmax: peak velocity of tricuspid regurgitation; NA: not applicable.

**Table 3:** Cox proportional hazard univariate analysis for dead at six months in 142 dogs with myxomatous mitral valve disease.

Predictors	Hazard Ratio	95% CI	Chi Square	*p*-Value
Age (months)	1.008	1.002–1.013	7.960	0.005
Weight (kg)	1.028	0.999.0–1.057	3.514	0.061
Sex—Male	1.152	0.770–1.724	0.474	0.491
Breed—Pure breed	0.815	0.547–1.213	1.016	0.313
Cardiac treatment Vs. No Treatment	2.001	1.235–3.241	7.946	0.005
Mixed treatment/disease Vs. No Treatment	1.828	0.886–3.770	2.667	0.1024
Other treatment/disease Vs. No Treatment	0.989	0.583–1.678	0.002	0.966
ACVIM stage C + D	1.870	1.212–2.886	7.999	0.005
AF	3.896	1.213–12.514	5.218	0.022
PH	1.876	0.998–3.526	3.813	0.051
Urea (mg/dL)	1.017	1.007–1.026	11.893	<0.001
Total protein (g/L)	1.016	0.988–1.046	1.246	0.264
Albumin (g/L)	1.044	0.986–1.105	2.209	0.137
Creatinine (mg/dL)	1.600	0.989–2.589	3.666	0.055
WBC (10^3^/μL)	1.043	1.007–1.080	5.527	0.019
RBC (10^6^/μL)	0.927	0.744–1.155	0.458	0.498
Hemoglobin (g/dL)	0.960	0.873–1.055	0.733	0.392
Hematocrit (%)	0.980	0.948–1.012	1.529	0.216
RDW (%)	1.245	1.103–1.405	12.609	<0.001
MCV (fL)	0.971	0.925–1.019	1.419	0.234
Platelet (10^3^/μL)	1.001	1.000–1.002	1.913	0.167
LA/Ao	2.108	1.488–2.987	17.582	<0.001
LVDDn	2.053	1.115–3.779	5.330	0.021
LVSDn	2.949	1.182–7.353	5.380	0.020
FS (%)	0.998	0.977–1.021	0.022	0.883
E-max (cm/s)	1.008	1.003–1.014	8.802	0.003
TR Vmax (m/s)	1.559	1.117–2.177	6.797	0.009

ACVIM: American College of Veterinary Internal Medicine; AF: atrial fibrillation; PH: pulmonary hypertension; WBC: white blood cell; RBC: red blood cell; RDW: red cell distribution width; MCV: mean corpuscular volume; LA/Ao: left atrial to aortic root diameter ratio; LVDDn: left ventricular diastolic diameter normalized for body weight; LVSDn: left ventricular systolic diameter normalized for body weight; FS: fractional shortening; E-max: trans-mitral peak E-wave velocity; TR Vmax: peak velocity of tricuspid regurgitation.

**Table 4:** Final multivariable model for all-cause mortality at six months.

Predictors	Hazard Ratio	95% CI	Chi Square	*p*-Value
Urea (mg/dL)	1.016	1.005–1.026	9.000	0.003
WBC (10^3^/μL)	1.043	1.004–1.083	4.734	0.030
RDW (%)	1.203	1.045–1.384	6.624	0.010
LA/Ao	1.892	1.232–2.905	8.478	0.004

WBC: white blood cell; RDW: red cell distribution width; LA/Ao: left atrial to aortic root diameter ratio.

## Data Availability

The data presented in this study are available on request from the corresponding author.

## Appendix A

**Table A1:** Type of treatment and drugs administered to 146 dogs with myxomatous mitral valve disease.

Therapy/Drugs	Number (%)
Cardiac treatment	47 (32.2)
Loop diuretics	36 (24.7)
ACE-Is	32 (21.9)
Pimobendan	26 (17.8)
Spironolactone	7 (4.8)
Digoxin	1 (0.7)
Mixed treatment	13 (8.9)
ACE-Is	10 (6.8)
Pimobendan	6 (4.1)
Loop diuretics	6 (4.1)
Steroids	6 (4.1)
Antibiotics	2 (1.4)
Anticonvulsants	2 (1.4)
Hepatoprotective drugs	2 (1.4)
NSAIDs	1 (0.7)
Antacids-Antiemetics	1 (0.7)
Vitamins-Other supplements	1 (0.7)
Other treatments	21 (14.4)
Antibiotics	4 (2.7)
Anticonvulsants	4 (2.7)
Steroids	2 (1.4)
Antacids-Antiemetics	2 (1.4)
Propentofylline	2 (1.4)
Levothyroxine	2 (1.4)
Hepatoprotective drugs	2 (1.4)
NSAIDs	1 (0.7)
Gabapentin	1 (0.7)
Trilostane	1 (0.7)
Vitamins-Other supplements	1 (0.7)
Antiandrogen	1 (0.7)
Phenylpropanolamine	1 (0.7)
No therapy	65 (44.5)

ACE-Is: angiotensin-converting enzyme inhibitors; NSAIDs: non-steroidal anti-inflammatory drugs.
